# Functional Outcomes of Difficult Primary Total Knee Arthroplasty Assessed by the Bristol Knee Score at Six Months: A Tertiary Care Center Experience

**DOI:** 10.7759/cureus.110829

**Published:** 2026-06-14

**Authors:** Jeevan George, George Sonu, Gokul Gopan, Sudhir R

**Affiliations:** 1 Department of Orthopaedics and Traumatology, Government Medical College, Thiruvananthapuram, Thiruvananthapuram, IND; 2 Department of Orthopaedics, Government Medical College, Thiruvananthapuram, Thiruvananthapuram, IND

**Keywords:** bristol knee score, difficult primary knee arthroplasty, osteoarthritis knee, postoperative outcomes, quality of life

## Abstract

Objective: This study aimed to evaluate the functional outcomes of difficult primary knee arthroplasty performed at a tertiary care center using the Bristol Knee Score.

Methodology: This study included patients attending the outpatient department (OPD) at Government Medical College, Thiruvananthapuram, who had undergone difficult primary total knee arthroplasty (TKA) six months prior. Informed consent was obtained from all participants. A minimum of 42 eligible patients were included in the study. After obtaining consent, a detailed clinical history and physical examination were conducted. Postoperative assessment included evaluation of follow-up anteroposterior (AP) and lateral radiographs of the knee. Functional outcomes were assessed using the Bristol Knee Score.

Results: The study demonstrated significant improvement in functional outcomes following surgery. Preoperatively, all patients had poor knee scores, with a mean Bristol Knee Score of 44.3±2.1. At six months postoperatively, the mean score improved significantly to 85.6±5.6. Among the participants, 17 (40.5%) patients achieved a good knee score, while 25 (59.5%) patients achieved an excellent knee score. These findings highlight the effectiveness of TKA in improving knee function and overall quality of life in patients with severe knee pathology.

Conclusion: No significant correlation was observed between functional outcomes and variables such as age, sex, socioeconomic status, comorbidities, body mass index (BMI), duration of surgery, or length of hospital stay. These findings suggest that TKA provides favorable outcomes across diverse patient groups.

## Introduction

Total knee replacement (TKR) is a reliable and well-established procedure for symptomatic relief and functional restoration in patients with end-stage osteoarthritis of the knee [1]. Difficult primary total knee arthroplasty (TKA) refers to first-time arthroplasty procedures that present technical challenges due to severe deformities, bone loss, soft tissue contractures, multiple comorbidities, or previous surgeries [2].

Primary TKA generally provides dependable and reproducible long-term outcomes. However, several patient-specific and local knee factors may increase surgical complexity [3]. Common challenging scenarios include patients with multiple prior surgical incisions, significant coronal plane deformities, genu recurvatum, stiff knees, extra-articular deformities, previous osteotomies around the knee, and chronic patellar dislocation [4].

Management of such cases requires careful evaluation of patient-related factors, meticulous preoperative planning, and an understanding of documented outcomes. Surgeons should adopt a systematic, evidence-based approach to managing difficult primary TKA cases, guided by the literature relevant to each specific clinical situation [5].

In contrast to Western populations, patients with osteoarthritis in India, particularly in Kerala, often present late with advanced deformities after exhausting traditional or alternative treatment modalities. As a result, many become candidates for difficult primary TKA. Inadequate management of these complex cases may lead to significant physical, psychological, and financial morbidity and, in rare instances, mortality [6].

Most available literature focuses on Western populations or patients from North India. Limited data are available from South India, particularly Kerala, where demographic characteristics and lifestyle factors differ substantially. Therefore, evaluating the factors contributing to difficult primary TKA and identifying the predictors of favorable functional outcomes in the Kerala population are essential for developing region-specific management protocols and improving long-term quality of life. Therefore, the primary objective of this study was to evaluate functional outcomes following difficult primary TKA using the Bristol Knee Score at six months postoperatively. The secondary objective was to assess the association of demographic and clinical variables, including age, sex, socioeconomic status, comorbidities, body mass index (BMI), duration of surgery, and length of hospital stay, with postoperative functional outcomes.

## Materials and methods

Study design and setting

This prospective cohort study was conducted in the Department of Orthopaedics at Government Medical College, Thiruvananthapuram, in Thiruvananthapuram, India, over a period of 18 months after obtaining approval from the institute's Institutional Research Committee (approval number: IRC/PG/260/2023/MCT). Patients were prospectively enrolled at the time of surgery, and baseline demographic and clinical variables were recorded at enrollment. Outcome assessment using the Bristol Knee Score [7] was performed at the six-month postoperative follow-up visit.

Study population

The study population comprised patients who had undergone difficult primary TKA for severe deformities, including valgus or varus deformity ≥20°, bone loss, fixed flexion contracture, multiple comorbidities, or prior surgeries involving the knee. Patients with degenerative or inflammatory arthritis affecting unilateral or bilateral knees were eligible for inclusion. All patients meeting the inclusion criteria and providing informed consent were enrolled consecutively until the required sample size was achieved.

Sample size calculation

As the primary objective of the study was to evaluate functional outcomes using the Bristol Knee Score [7] at six months following difficult primary TKA, all consecutive eligible patients fulfilling the inclusion criteria during the study period were included. A total of 42 patients completed the six-month follow-up assessment and were included in the final analysis. The study should be considered exploratory, and the findings interpreted in light of the relatively small sample size. The eligibility criteria for patient selection, including both inclusion and exclusion criteria, are summarized in Table 1.

**Table 1:** Inclusion and exclusion criteria for patient selection

Inclusion criteria	Exclusion criteria
Patients aged ≥18 years with degenerative or inflammatory arthritis involving unilateral or bilateral knees	Patients who declined to provide consent
Patients who underwent difficult primary total knee arthroplasty	Prior surgery for loco-regional tumors or metastasis involving the same joint
Severe valgus deformity (≥20°)	Neuropathic arthropathy
Severe varus deformity (≥20°)	Conditions preventing postoperative mobilization
History of prior knee surgeries or previous surgical incisions around the knee	Septic arthritis of the knee joint
Presence of multiple comorbidities	Deformity or disability of the ipsilateral hip joint
Fixed flexion contracture of the knee	Deformity or disability of the ipsilateral ankle joint
Presence of periarticular bone loss	-

Data collection procedure

After obtaining informed consent, a detailed clinical history was recorded, and a comprehensive physical examination was performed. Follow-up radiographs, including anteroposterior and lateral views of the knee, were assessed. Functional outcomes were evaluated at six months postoperatively using the Bristol Knee Score [7].

Baseline demographic and clinical variables, including age, sex, socioeconomic status, BMI, comorbidities, radiographic deformity, duration of surgery, and length of hospital stay, were recorded at enrollment and subsequently verified from medical records.

Radiographic knee angle assessment was performed using standard anteroposterior radiographs to evaluate the degree of varus or valgus deformity.

The Bristol Knee Score [7] was administered during the six-month follow-up visit by members of the treating orthopaedic team through direct patient assessment based on pain, function, and mobility parameters. Formal blinding of outcome assessors was not performed because of the observational nature of the study.

Data regarding implant type, surgical approach, use of stems or augments, patellar resurfacing, intraoperative blood loss, rehabilitation adherence, and surgeon experience were not collected as part of the study protocol and therefore were not included in the analysis.

All patients underwent standard postoperative rehabilitation according to institutional protocols, including early mobilization and supervised physiotherapy to improve range of motion and functional recovery.

Statistical analysis

A comprehensive statistical analysis was conducted to evaluate functional outcomes following difficult primary TKA and to determine associations with demographic and clinical variables. The primary outcome measure was the Bristol Knee Score, assessed preoperatively and at six months postoperatively.

Continuous variables, including age, weight, height, radiographic knee angle, duration of surgery, and length of hospital stay, were summarized using mean±standard deviation (SD) or median with interquartile range (IQR), as appropriate. Categorical variables, such as sex, socioeconomic status, comorbidities, BMI categories, and functional outcome categories, were presented as frequencies and percentages.

The Wilcoxon signed-rank test was used to compare preoperative and postoperative Bristol Knee Scores. Associations between functional outcomes and categorical variables were analyzed using the chi-squared test. A p-value of <0.05 was considered statistically significant. Statistical analyses were performed using jamovi (Version 2.5.3).

## Results

A total of 42 patients undergoing difficult primary TKA were included in the study. The age distribution showed that 19 (45.2%) patients were aged ≤60 years and 23 (54.8%) were >60 years. The mean age was 61.2±7.1 years (range: 48-77 years). All patients presented with severe preoperative deformity, with a mean radiographic knee angle of 24.9±3.2°. The mean preoperative Bristol Knee Score was 44.3±2.1, indicating uniformly poor baseline function. The mean operative time was 1.97±0.37 hours; 19 (45.2%) patients underwent procedures lasting <2 hours, while 23 (54.8%) had operative times >2 hours. The mean length of hospital stay was 7.6±0.8 days, with 18 (42.9%) patients hospitalized for ≤1 week and 24 (57.1%) for >1 week (Table 2).

**Table 2:** Descriptive statistics of radiological, operative, and functional outcome variables among patients undergoing difficult primary total knee arthroplasty (n=42)

Variables	Mean±SD	Range	Median	IQR
X-ray knee angle (°)	24.9±3.2	20-30	25	25-25
Preoperative Bristol Knee Score	44.3±2.1	40-49	44	42.8-46
Duration of surgery (hours)	1.97±0.37	1.17-2.5	2	1.8-2.3
Length of hospital stay	7.6±0.8	5-10	8	7-8
Bristol Knee Score at 6 months	85.6±5.6	75-95	86	81.75-89

All 42 patients were evaluated preoperatively and at six months postoperatively. At six months, the mean Bristol Knee Score improved significantly to 85.6±5.6 (median: 86; IQR: 81.75-89), compared to the preoperative median of 44 (IQR: 42.8-46). The Wilcoxon signed-rank test demonstrated a statistically significant improvement in functional outcome (z=5.649; p<0.001), indicating that all patients (42, 100%) experienced postoperative functional gain (Table 3).

**Table 3:** Comparison of preoperative and six-month postoperative Bristol Knee Scores using the Wilcoxon signed-rank test (n=42)

Outcomes	Bristol Knee Score				Wilcoxon signed-rank test	
	Mean±SD	Range	Median	IQR	z	p
Preoperatively	44.3±2.1	40-49	44	42.8-46	5.649	<0.001
Postoperatively at 6 months	85.6±5.6	75-95	86	81.75-89		

Regarding functional outcome categories, 17 (40.5%) patients achieved good outcomes, and 25 (59.5%) achieved excellent outcomes at six months. No statistically significant association was observed between six-month functional outcome and demographic or clinical variables. Age group (≤60 vs. >60 years; p=0.845) and sex (male: 10 (23.8%); female: 32 (76.2%); p=0.482) were not significantly associated with outcome. Most patients were below the poverty line (33, 78.6%) and had at least one comorbidity (34, 81%); however, neither socioeconomic status (p=0.784) nor comorbidity status (p=0.849) significantly influenced functional outcome. Regarding BMI, nine (21.4%) patients had normal BMI, 30 (71.4%) were overweight, and three (7.1%) were obese; BMI category was not significantly associated with outcome (p=0.110). Similarly, operative duration (<2 vs. >2 hours; p=0.845) and length of hospital stay (≤1 vs. >1 week; p=0.414) showed no significant association with six-month functional outcome. Overall, none of the evaluated variables demonstrated a statistically significant relationship with postoperative functional status (Table 4).

**Table 4:** Association between demographic and clinical variables and functional outcome at six months (n=42)

Variables	Functional outcomes		Total	Chi-square (df)	Cramer's V	P-value
	Good	Excellent				
Age				0.04 (1)	0.03	0.845
≤60 years	8 (47.1%)	11 (44%)	19 (45.2%)			
>60 years	9 (52.9%)	14 (56%)	23 (54.8%)			
Sex				0.50 (1)	0.11	0.482
Male	5 (29.4%)	5 (20%)	10 (23.8%)			
Female	12 (70.6%)	20 (80%)	32 (76.2%)			
Socioeconomic status				0.08 (1)	0.04	0.784
Above the poverty line	4 (23.5%)	5 (20%)	9 (21.4%)			
Below the poverty line	13 (76.5%)	20 (80%)	33 (78.6%)			
Comorbidities				0.04 (1)	0.03	0.849
Yes	14 (82.4%)	20 (80%)	34 (81%)			
No	3 (17.6%)	5 (20%)	8 (19%)			
Body mass index				4.41 (2)	0.32	0.110
Normal	2 (11.8%)	7 (28%)	9 (21.4%)			
Overweight	15 (88.2%)	15 (60%)	30 (71.4%)			
Obese	0 (0%)	3 (12%)	3 (7.1%)			
Duration of surgery				0.04 (1)	0.03	0.845
<2 hours	8 (47.1%)	11 (44%)	19 (45.2%)			
>2 hours	9 (52.9%)	14 (56%)	23 (54.8%)			
Hospital stay				0.67 (1)	0.13	0.414
≤1 week	6 (35.3%)	12 (48%)	18 (42.9%)			
>1 week	11 (64.7%)	13 (52%)	24 (57.1%)			

## Discussion

The present prospective cohort study evaluated functional outcomes following difficult primary TKA in patients with severe deformities, prior surgeries, comorbidities, or bone loss, using the Bristol Knee Score as the primary outcome measure. A statistically significant improvement in functional status was observed at six months postoperatively, with the mean Bristol Knee Score increasing from 44.3±2.1 preoperatively to 85.6±5.6 postoperatively (p<0.001). A majority of patients achieved favorable outcomes, with 40.5% attaining good scores and 59.5% achieving excellent functional scores.

These findings are consistent with previous literature demonstrating substantial functional improvement following TKA in patients with severe deformities. Karachalios et al. [8] reported significant postoperative functional restoration in patients undergoing TKA for severe varus and valgus deformities, concluding that appropriate deformity correction and soft tissue balancing yield favorable outcomes. Similarly, Ranawat et al. [9] emphasized that even complex primary TKA cases can achieve outcomes comparable to standard TKA when meticulous surgical technique is employed. Although different scoring systems, such as the Knee Society Score (KSS), were used in these studies, the magnitude of functional improvement aligns closely with that observed in the present cohort.

Age was not significantly associated with functional outcomes (p=0.845). Patients aged ≤60 years and those >60 years demonstrated comparable postoperative improvements. This finding is consistent with Elmallah et al. [10], who reported that although older patients may present with lower baseline function, postoperative functional gains are similar across age groups. Likewise, Lützner et al. [11] concluded that age alone should not be considered a contraindication to TKA, as significant improvements can be achieved when comorbidities are adequately optimized.

Sex was also not significantly associated with postoperative outcomes (p=0.482). Despite a higher proportion of female participants, both sexes achieved comparable rates of good and excellent outcomes. Bourne et al. [12] similarly reported no consistent long-term differences in objective functional outcomes between male and female patients following TKA. Although some studies suggest that females may report lower subjective satisfaction scores, objective functional recovery appears comparable across genders.

Socioeconomic status did not significantly influence functional outcomes (p=0.784). This finding suggests that in a tertiary care government setting, standardized surgical care and postoperative rehabilitation may reduce disparities related to socioeconomic background. Singh et al. [13] also observed that while socioeconomic factors may influence access to surgical care, postoperative functional improvement is primarily determined by clinical and surgical factors rather than income status alone.

The presence of comorbidities was not significantly associated with functional outcomes (p=0.849). Although a substantial proportion of patients had coexisting medical conditions, satisfactory functional recovery was achieved. Elmallah et al. [14] similarly reported that while comorbidities may increase perioperative risk, they do not necessarily compromise mid-term functional recovery when appropriately managed. Nevertheless, other studies indicate that poorly controlled comorbidities may delay early recovery, underscoring the importance of thorough preoperative optimization.

BMI was not significantly associated with functional outcomes (p=0.110). Although obesity has traditionally been regarded as a risk factor for inferior outcomes and complications, contemporary evidence supports comparable functional gains. Kerkhoffs et al. [15] concluded that obese patients can achieve improvements similar to non-obese patients following TKA, despite a slightly higher risk of complications. Foran et al. [16] likewise demonstrated significant postoperative functional gains among obese individuals, even if absolute functional scores may be marginally lower.

Duration of surgery did not significantly affect functional outcomes (p=0.845). While prolonged operative time may reflect surgical complexity and increase perioperative risk, it does not appear to determine functional recovery at six months independently. Namba et al. [17] reported that extended operative time is more strongly associated with infection risk rather than long-term functional impairment. Similarly, Peersman et al. [18] noted that operative duration alone does not significantly influence postoperative knee function.

Length of hospital stay was also not significantly associated with functional outcomes (p=0.414). Enhanced recovery protocols and standardized rehabilitation pathways may explain the lack of correlation between hospitalization duration and functional recovery. Kehlet and Wilmore [19] highlighted that early mobilization and optimized perioperative care can reduce hospital stay without compromising outcomes. Husted et al. [20] further demonstrated that shorter hospitalization under fast-track protocols does not adversely affect functional results.

Overall, the findings of this study support existing evidence that difficult primary TKA can yield excellent short-term functional outcomes when appropriate surgical techniques and structured rehabilitation protocols are employed. Demographic variables, such as age and sex, as well as clinical variables, including BMI, comorbidities, operative duration, and length of hospital stay, did not significantly influence short-term functional recovery in this cohort.

Limitations

This study has several limitations that should be acknowledged. First, it was conducted at a single tertiary care center with a relatively small sample size (n=42), which may limit the generalizability of the findings and reduce the statistical power to detect subtle associations between clinical variables and functional outcomes. Second, the follow-up period was limited to six months, restricting the evaluation to short-term outcomes. Long-term implant survival, late complications, and sustained functional improvement were not assessed. Third, the absence of a control group undergoing routine primary TKA precluded direct comparative analysis. Functional outcomes were assessed solely using the Bristol Knee Score, without the incorporation of additional validated patient-reported outcome measures. Moreover, detailed radiological parameters, such as component alignment and implant positioning, were not systematically correlated with functional results. In addition, variations in surgical technique, implant selection, surgeon experience, and perioperative management protocols were not specifically analyzed, which may affect the external reproducibility of the findings. Furthermore, data regarding implant type, surgical approach, use of stems or augments, patellar resurfacing, intraoperative blood loss, surgeon experience, and adherence to postoperative rehabilitation protocols were not systematically collected. Therefore, the potential influence of these variables on functional outcomes could not be evaluated, and residual confounding cannot be excluded. Finally, as an observational study, causal relationships cannot be established. Unmeasured confounding factors, including adherence to postoperative rehabilitation protocols and psychosocial influences, may also have affected recovery outcomes.

## Conclusions

This study demonstrated no significant association between functional outcomes following difficult primary TKA and variables such as age, sex, socioeconomic status, comorbidities, BMI, duration of surgery, or length of hospital stay. These findings indicate that TKA is an effective intervention across diverse patient groups, irrespective of demographic and selected clinical factors. However, these findings should be interpreted cautiously due to the relatively small sample size and limited statistical power, which may have reduced the ability to detect subtle associations. Additionally, the short follow-up period restricts interpretation to early postoperative outcomes. Nevertheless, variations reported in previous studies underscore the need for further large-scale, multicenter research to identify additional determinants of functional recovery and to validate these findings in broader and more heterogeneous populations.
